# Changes of Subfoveal Choroidal Thickness after Cataract Surgery: A Meta-Analysis

**DOI:** 10.1155/2018/2501325

**Published:** 2018-11-12

**Authors:** Shaoxue Zeng, Chen Liang, Yueqing He, Yingying Chen, Qing Zhao, Shuiping Dai, Fengrui Cheng, Junjun Zhang, Xiaoshuang Jiang

**Affiliations:** ^1^Department of Ophthalmology, West China Hospital, Sichuan University, Chengdu 610041, China; ^2^West China School of Medicine, Sichuan University, Chengdu 610041, China

## Abstract

**Purpose:**

To investigate the effect of cataract surgery on subfoveal choroid thickness (SFCT) using enhanced-depth imaging optical coherence tomography (EDI-OCT).

**Materials and Methods:**

Relevant publications were searched systematically through various databases from inception to March 2018. The unit of choroidal thickness measurements is micrometers. Studies comparing SFCT before and after cataract surgery were retrieved. All qualified articles were analyzed using RevMan 5.3.

**Results:**

A total of 13 studies with 802 eyes from 646 patients were identified for inclusion. There was a significant increase of SFCT at 1 week (MD = 6.62, 95% CI: 1.20–12.05, *P*=0.02, I^2^ = 0%), 1 month (MD = 8.30, 95% CI: 3.20–13.39, *P*=0.001, I^2^ = 0%), and 3 months (MD = 8.28, 95% CI: 1.84–14.73, *P*=0.01, I^2^ = 0%) after cataract surgery. In subgroup analysis, SFCT in Asians and patients without nonsteroidal anti-inflammatory drugs (NSAIDs) in postoperative medication was significantly thicker (*P* < 0.05). No statistically significant increase of SFCT was found in diabetic mellitus (DM) patients for 1 day (*P*=0.89), 1 week (*P*=0.59), 1 month (*P*=0.52), and 3 months (*P*=0.42) after cataract surgery.

**Conclusions:**

This meta-analysis suggested that SFCT increased since 1 week after the cataract surgery and the increase lasted for at least 3 months. Asians and patients without NSAIDs in postoperative medication were more likely to have a thicker SFCT after cataract surgery, whereas DM patients were less likely to increase in SFCT.

## 1. Introduction

Cataract was featured by the opacification of the crystalline lens and was the leading cause of blindness all over the world [1]. According to the World Health Organization (WHO), there was an estimated 180 million visually disabled people worldwide, among which, 46% of them were the result of cataracts [2]. Phacoemulsification and intracapsular lens implantation were the most commonly performed eye surgeries to correct the visual loss and restore the vision for patients with cataract [3]. However, complications of cataract surgery on posterior segment of the eye were estimated in many studies. Pseudophakic macular edema and progression of diabetic retinopathy were the most common adverse effects on retina after cataract surgery [4, 5]. Specifically, studies have also shown the relation between cataract surgery and the onset of age-related macular degeneration (AMD) [6]. In rodent studies, it was reported that lens extraction could trigger proinflammatory gene expression and protein response in the posterior segment of the eyes [7]. An other study in mice has also shown that partial lens extraction resulted in blood-retinal barrier breakdown [8]. These findings implied the possible influence of cataract surgery on posterior segment of eyes.

It was reported that macular or retinal thickness increased after cataract surgery [9]. Choroid was a vascular structure in close relation to the retina in the eye, and changes in choroid thickness might also be predicted. Enhanced-depth imaging (EDI) OCT has become a widely used way for clinical and research applications, and we could use this noninvasive method to detect the full thickness of the choroid in detail [10].

Several studies have been conducted to assess the influence of cataract surgery to subfoveal choroid thickness (SFCT). Studies [11–20] reported that most patients with senile cataract were expected to maintain increased SFCT for several months after cataract surgery, suggesting the inflammatory response of the surgery or the onset of AMD, which was originated from the choroid layer. However, several studies [21–23] hold a different view that no significant increase was detected in SFCT after the surgery. Therefore, we performed this meta-analysis of the available published literatures to explicate the relationship between SFCT and cataract surgery.

## 2. Methods

### 2.1. Literature Search

We searched the electronic databases such as PubMed, Embase, Web of Science, and China National Knowledge Infrastructure (CNKI) until March 2018 for all the relevant literatures using the search terms “choroid thickness” and “cataract surgery” or “phacoemulsification” or “cataract extraction”. The computer search was supplemented with manual search by checking the reference lists of all retrieved studies and reviews to include eligible studies. There was no language restriction.

### 2.2. Inclusion and Exclusion Criteria

The inclusion criteria were as follows: (1) studies recruiting patients who experienced cataract extraction and intraocular lens implant; (2) SFCT before and after cataract surgery was measured; (3) data including mean differences (MDs) with 95% confidence intervals (CIs) were recorded. Abstracts from conferences, editorials, letters, review articles, full texts without raw data available for retrieval, and duplicate publications were excluded. If there were studies with overlapped patients, small sample studies were excluded.

### 2.3. Quality Assessment of the Studies

The methodological quality of cohort study was assessed using the modified Newcastle–Ottawa Scale (NOS) [24]. A total of eight items were categorized into three dimensions, namely, selection, comparability, and outcome. Each item in selection and outcome was awarded a maximum of one star while the item of comparability could score two stars; thus, the range of NOS is zero to nine. Studies with a score of 6 or higher were considered high quality.

### 2.4. Data Extraction

Two reviewers screened and extracted the data independently. The third reviewer made the final decision if there was any inconsistency. The following data were extracted from literatures: first author, year of publication, country, ethnicity, number of patients and studied eyes, inclusion and exclusion criteria, characteristics of study subjects, and SFCT with 95% CIs at different time intervals (1 day before the surgery and 1 day, 1 week, 1 month, and 3 months after the surgery). All the measurements of choroidal thickness are in micrometers, and the data of patients with diabetic retinopathy (DR) were also recorded.

### 2.5. Statistical Analysis

This meta-analysis compared the SFCT at different time intervals after the cataract surgery with the initial baseline value. We used the Cochrane Review Manager (software version 5.3) to analysis the data. The WMD was determined for SFCT at different time intervals with a 95% CI. The I^2^ statistic was applied to assess heterogeneity between studies [25]. A random effects model was used when the heterogeneity ≥ 50%, or a fixed effects model was applied. Subgroup analysis was performed according to ethnicity, surgery machine, sample numbers, medication, OCT machine, and quality control and if patients were diagnosed with diabetes mellitus. Sensitivity analysis was performed by dropping out each study one by one. Funnel plots were used to evaluate the publication bias. If *P* value in our meta-analysis was less than 0.05, the results were thought to be significant.

## 3. Results

**Figure 1:**
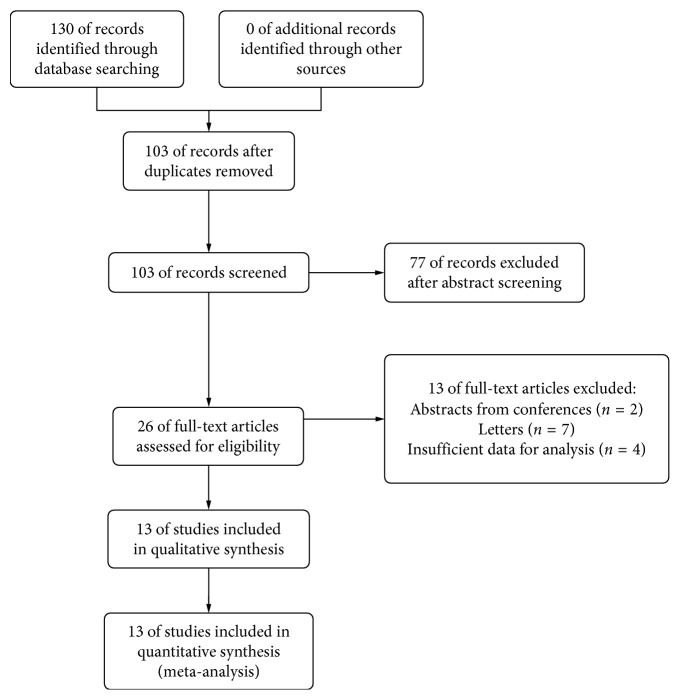
Flow diagram of the selection process in the meta-analysis. SFCT: postoperative 1 d versus preoperative.

A total of 130 studies were initially identified, of which 27 were duplicates and 77 were rejected based on titles and abstracts. In further full-text reading, we excluded 7 letters, 2 conference papers, and 4 studies without efficient data. At last, a total of 13 studies including 802 eyes from 646 patients were enrolled in the final analysis. The flow diagram of the search procedure and results are shown in Figure 1 and characteristics of included studies are listed in Table 1. The published time of these studies ranges from July 2014 to November 2017. There were 12 prospective cohort studies and 1 retrospective study. The ethnicity of patients in 9 studies was Caucasians, and the ethnicity of the other 4 was Asians. In our meta-analysis, we did not drop out studies that were conducted in patients with diabetic mellitus (DM), we included 67 eyes from patients with DM, and all the other patients were non-DM patients. The surgery methods in these studies were all phacoemulsification. Postoperative treatment consisted of antibiotics in all the studies but differed in the use of nonsteroidal anti-inflammatory drugs (NSAIDs). Of all the patients, no other complications developed except for 3 patients who developed macular edema, who were also included in our study. Distances between the RPE and choroidal-scleral interface were measured manually to be SFCT. However, difference may lie in if the OCT machines were different, if they had used 100 times scans and if the measurements were conducted in the same time of a day (Table 1).

### 3.1. SFCT: Postoperative 1 d versus Preoperative

**Figure 2:**
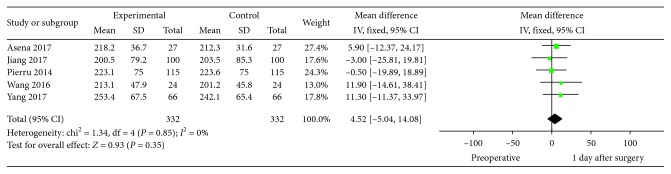
SFCT: postoperative 1 d versus preoperative.

A total of 5 studies including 332 eyes provided detailed information on SFCT 1 day after cataract surgery. SFCT before and 1 day after cataract surgery was not significantly different (MD = 4.52, 95% CI: −5.04–14.08, *P*=0.35, I^2^ = 0%).  Figure 2 shows the detailed information of these 5 studies.

### 3.2. SFCT: Postoperative 1 w versus Preoperative

**Figure 3:**
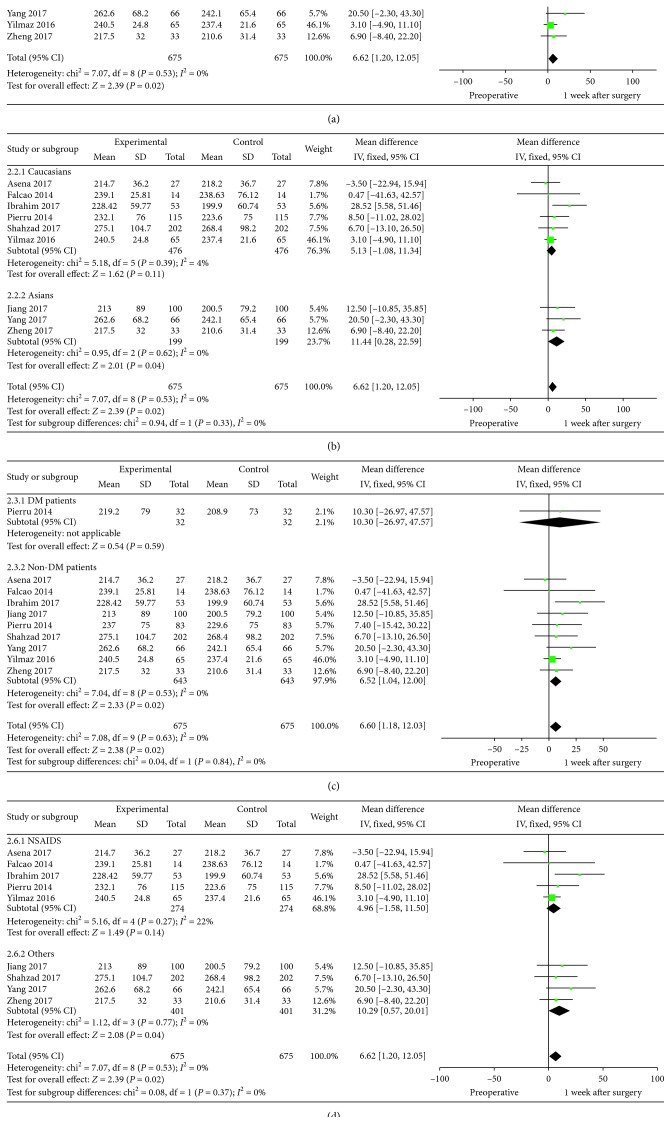
SFCT and subgroup analysis (ethnicity, if they were DM patients and if NSAIDs were included in the postoperative medication): postoperative 1 w versus preoperative.

A total of 9 studies including 675 eyes provided detailed information on SFCT 1 week after cataract surgery. The SFCT 1 week after the surgery was thicker compared with the baseline value, and the difference was statistically significant (MD = 6.62, 95% CI: 1.20–12.05, *P*=0.02, I^2^ = 0%). Subgroup analysis presented that 1 week after cataract surgery, we could also see increase of SFCT in Asians (MD = 11.44, 95% CI: 0.28–22.59, *P*=0.04, I^2^ = 0%) and patients without NSAIDs as postoperative medication (MD = 10.29, 95% CI: 0.57–20.01, *P*=0.04, I^2^ = 0%). A similar increase of SFCT could also be seen in groups with Alcon surgery machines, big samples, Heidelberg EDI-OCT machines, 100 times B-scans, and fixed scan time. For DM patients, SFCT (MD = 10.30, 95% CI: −26.97–47.57, *P*=0.59, I^2^ = 0%) was less likely to increase than patients without DM (MD = 6.52, 95% CI: 1.04–12.00, *P*=0.02, I^2^ = 0%).  Figure 3 shows the detailed information and subgroup analysis of these 9 studies.

### 3.3. SFCT: Postoperative 1 m versus Preoperative

**Figure 4:**
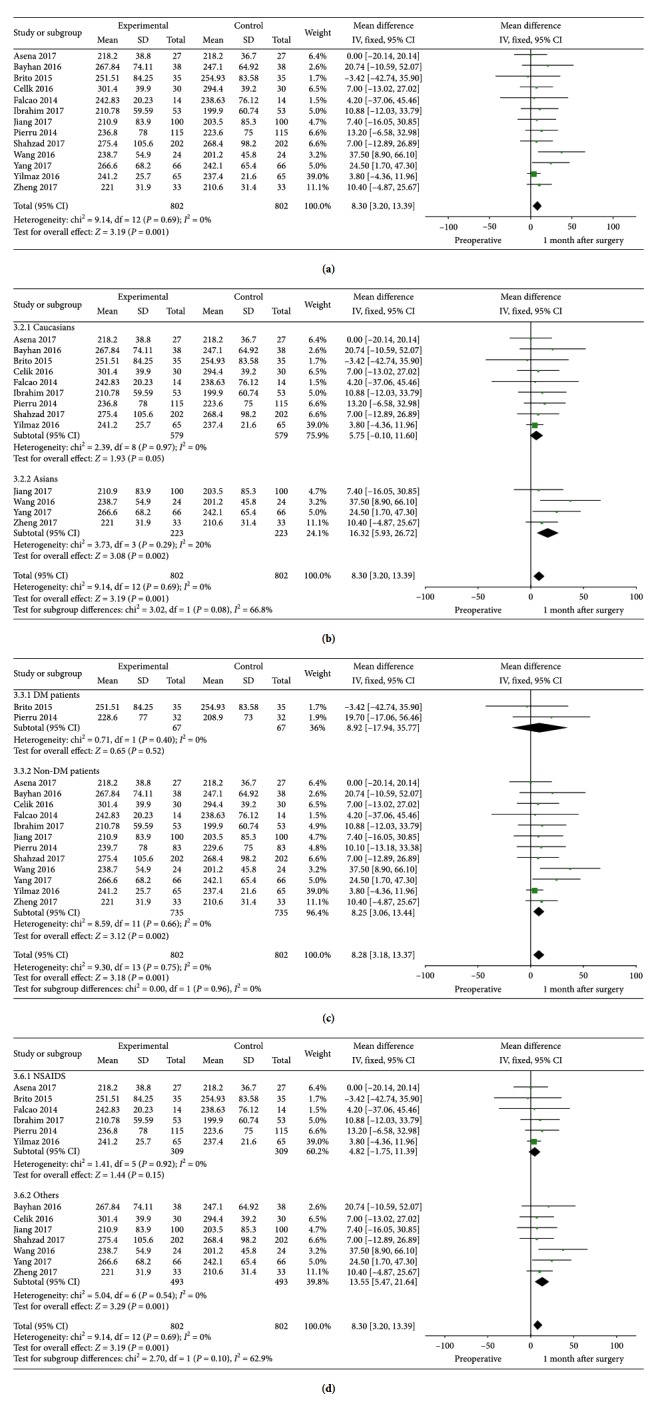
SFCT and subgroup analysis (ethnicity, if they were DM patients and if NSAIDs were included in the postoperative medication): postoperative 1 m versus preoperative.

All of the 13 studies provided detailed information on SFCT 1 month after cataract surgery. We found that SFCT at 1 month postoperatively was ∼8 *μ*m thicker than that of preoperatively, and there was a statistically significant difference (MD = 8.30, 95% CI: 3.20–13.39, *P*=0.001, I^2^ = 0%). Subgroup analysis presented that 1 month after cataract surgery, SFCT increased in Asians (MD = 16.32, 95% CI: 5.93–26.72, *P*=0.002, I^2^ = 20%), patients without NSAIDs in the postoperative medication (MD = 13.55, 95% CI: 5.47–21.64, *P*=0.001, I^2^ = 0%), patients who did not use Alcon as surgery machine (MD = 8.31, 95% CI: 2.87–13.76, *P*=0.003, I^2^ = 2%), patients whose averaged scans were 100 times (MD = 9.10, 95% CI: 0.75–17.46, *P*=0.03, I^2^ = 0%), and patients whose OCT scans were not obtained in the same time period of a day (MD = 11.60, 95% CI: 4.16–19.03, *P*=0.002, I^2^ = 0%) and the differences were statistically significant. Statistically significant results could also be detected in subgroups, no matter the sample capacity was big (>50) (MD = 7.37, 95% CI: 1.14–13.61, *P*=0.02, I^2^ = 0%) or small (<50) (MD = 10.16, 95% CI: 1.30–19.03, *P*=0.02, I^2^ = 0%). For DM patients, SFCT (MD = 8.92, 95% CI: −17.94–35.77, *P*=0.52, I^2^ = 0%) was less likely to increase than patients without DM (MD = 8.25, 95% CI: 3.06–13.44, *P*=0.002, I^2^ = 0%).  Figure 4 shows the detailed information and subgroup analysis of these 13 studies.

### 3.4. SFCT: Postoperative 3 m versus Preoperative

**Figure 5:**
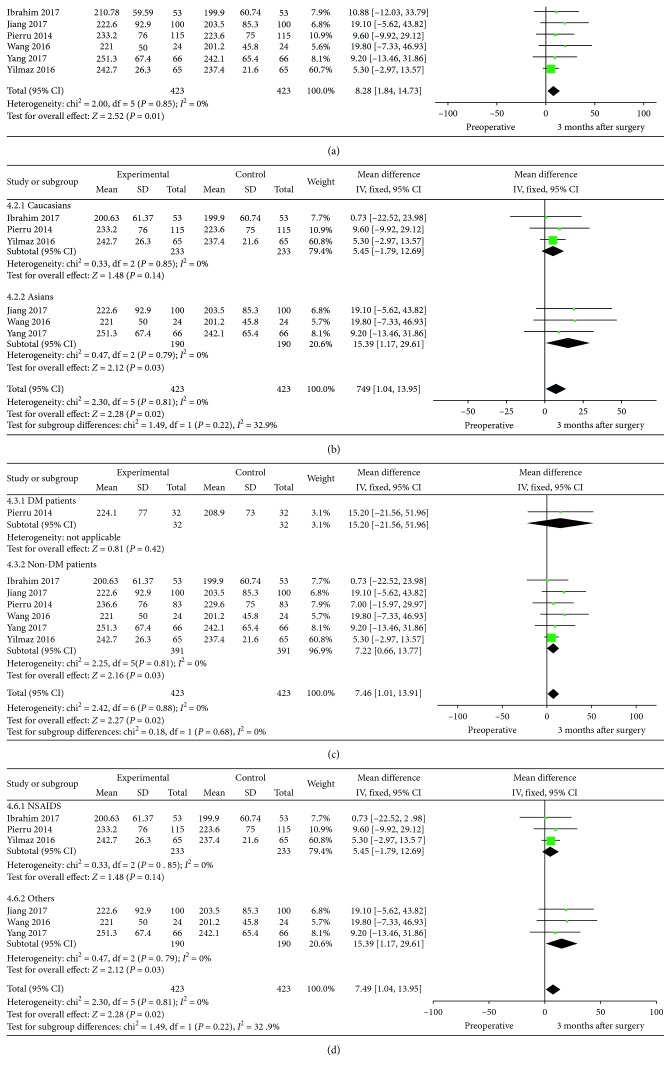
SFCT and subgroup analysis (ethnicity, if they were DM patients and if NSAIDs were included in the postoperative medication): postoperative 3 m versus preoperative.

A total of 6 studies including 423 eyes provided detailed information on SFCT 3 months after cataract surgery. We found that SFCT at 3 months postoperatively was ∼8 *μ*m thicker than that of preoperatively (MD = 8.28, 95% CI: 1.84–14.73, *P*=0.01, I^2^ = 0%). In subgroup analysis, there was statistically significant difference in the SFCT between Asians (MD = 15.39, 95% CI: 1.17–29.61, *P*=0.03, I^2^ = 0%) and Caucasians (MD = 5.45, 95% CI: −1.79–12.69, *P*=0.14, I^2^ = 0%). Statistically significant results could also be found in non-DM patients (MD = 7.22, 95% CI: 0.66–13.77, *P*=0.03, I^2^ = 0%), whereas in DM patients (MD = 15.20, 95% CI: −21.56–51.96, *P*=0.42), SFCT was not statistically significant. SFCT in patients who used the postoperative medication without NSAIDs (MD = 15.39, 95% CI: 1.17–29.61, *P*=0.03, I^2^ = 0%) was statistically thicker than those with NSAIDs.  Figure 5 shows the detailed information and subgroup analysis of these 6 studies.

### 3.5. SFCT: Subgroup Analysis in Non-DM Patients

Detailed information comparing the SFCT 1 day postoperatively with that of preoperatively in non-DM patients was found in 5 studies. No heterogeneity was found in any subgroup analysis. However, in the 9 studies which compare SFCT 1 week postoperatively and that of preoperatively, SFCT in Asians (MD = 11.44, 95% CI: 0.28–22.59, *P*=0.04, I^2^ = 0%) and patients who did not apply NSAIDs as postoperative medication (MD = 10.29, 95% CI: 0.57–20.01, *P*=0.04, I^2^ = 0%) was statistically thicker.

**Figure 6:**
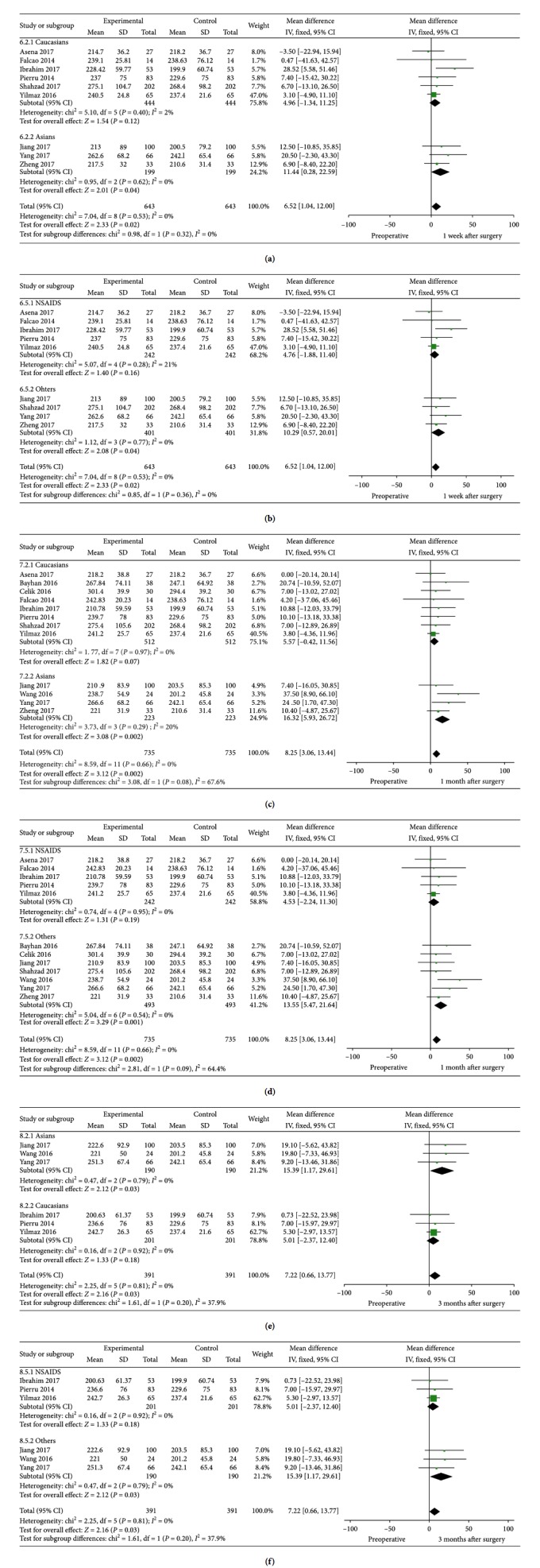
Subgroup analysis (ethnicity and if NSAIDs were included in the postoperative medication) in non-DM patients: postoperative 1 w versus preoperative, postoperative 1m versus preoperative, and postoperative 3 m versus preoperative.

Similar statistically significant results can be found in the 1 month subgroup analysis in Asians (MD = 16.32, 95% CI: 5.93–26.72, *P*=0.002, I^2^ = 0%) and patients who used the postoperative medication without NSAIDs (MD = 13.55, 95% CI: 5.47–21.64, *P*=0.001, I^2^ = 0%). In the 3 months subgroup, statistically significant results were also demonstrated in Asians (MD = 15.39, 95% CI: 1.17–29.61, *P*=0.03, I^2^ = 0%) and patients who did not apply NSAIDs as postoperative medication (MD = 15.39, 95% CI: 1.17–29.61, *P*=0.03, I^2^ = 0%).  Figure 6 shows the detailed information on subgroup analysis of all non-DM patients.

### 3.6. SFCT: Subgroup Analysis in the Different Use of NSAIDs

**Figure 7:**
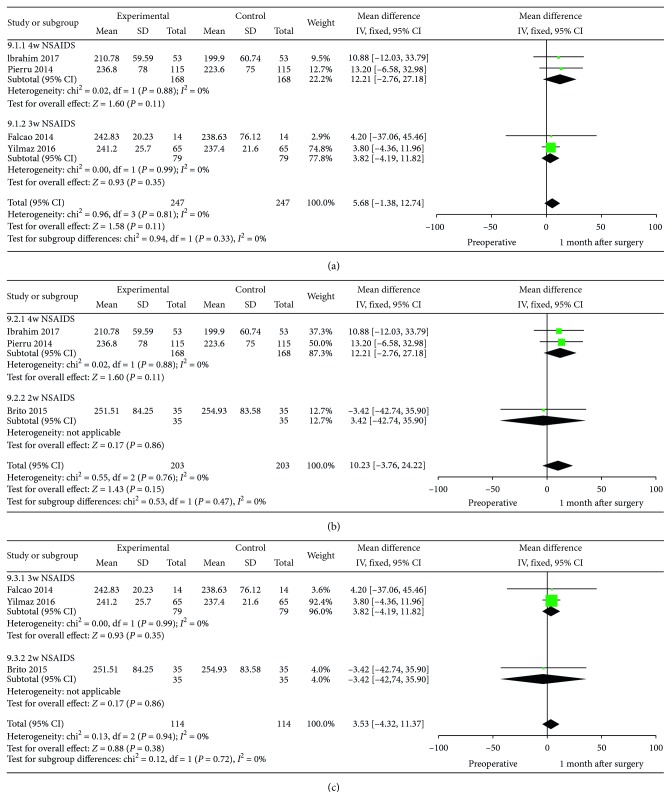
Subgroup analysis of different medication time of NSAIDs (4 w NSAIDs versus 3 w NSAIDs, 4 w NSAIDs versus 2 w NSAIDs, and 3 w NSAIDs versus 2 w NSAIDs): postoperative 1m versus preoperative.

In the 6 studies with the use of NSAIDs, detailed information about the medication time was found in 5 studies, containing 2 weeks in 1 study (MD = 12.21, 95% CI: −2.76–27.18, *P*=0.88, I^2^ = 0%), 3 weeks in 2 studies (MD = 3.82, 95% CI: −4.19–11.82, *P*=0.99, I^2^ = 0%), and 4 weeks in 2 studies (MD = –3.42, 95% CI: –42.74–35.90, *P*=0.76, I^2^ = 0%). No statistically significant difference was found among the 3 groups (Figure 7).

### 3.7. Publication Bias and Sensitivity Analysis

**Figure 8:**
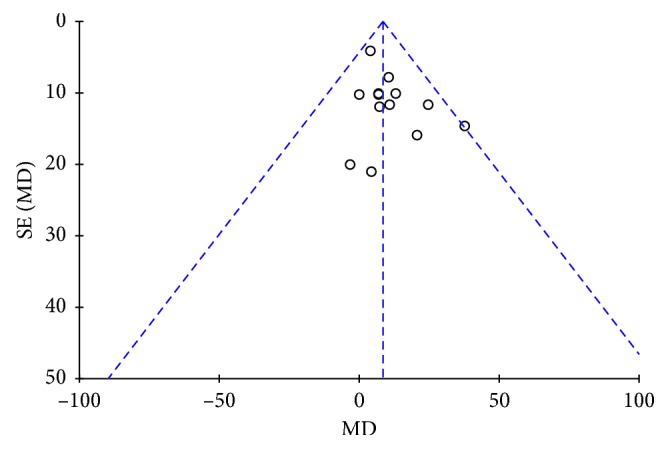
Publication bias.

No publication bias was found through the inverted funnel plot. As shown in Figure 8, the results were of stability because the results did not change significantly when dropping out each study one by one. Further sensitivity analyses were not conducted in the subgroup analysis because of the small sample sizes.

## 4. Discussion

In this meta-analysis, we reviewed 13 relevant studies, including a total of 802 eyes from 646 patients. The results from the group comparisons clearly demonstrated that the SFCT of patients was thicker at 1 week, 1 month, and 3 months postoperatively compared to the SFCT before the surgery. At 1 day postoperatively, the SFCT was not statistically significant comparing to baseline values because of the need of reaction time. At 1 week postoperatively, we could observe statistically significant difference, and the difference was even more obvious at 1 month after the surgery. The difference lasted to 3 months after the surgery. These results were statistically significant in accordance with some subgroup analyses.

The development of enhanced-depth imaging (EDI) OCT made it possible for doctors to detect choroid thickness [10]. Also, studies [26–28] held the idea that OCT was of good reliability, repeatability, and reproducibility to assess the choroid in detail. Studies have shown that SFCT has a greater chance to increase in male sex or patients with a thicker baseline SFCT [29].

Cataract surgery was capable of affecting the posterior segments of eyes in rodents and humans; especially, pseudophakic cystoid macular edema (PCME) was a common complication of cataract surgery [7, 30, 31]. Studies [11–20] showed an increase in SFCT after cataract surgery in humans, though the mechanism for the increase in the choroid thickness after cataract surgery remained unknown. What are the possible mechanisms? Animal study showed that extracapsular lens extraction could upregulate the expression of IL-1 and CCL2 genes in the neurosensory retina of C57BL/6 mice 30 minutes postoperatively and maintained for at least 2 weeks, which suggested that the surgery caused the acute inflammatory/injury response in posterior segments [7]. What is more, massive studies have observed that inflammatory disorders could increase the choroidal thickness in focal or systematical diseases such as uveitis, evanescent white dot syndrome, and chronic hepatitis C virus (HCV) infection [32–34]. With the inflammatory theory, we can explain that after cataract surgery, SFCT was significantly greater compared with baseline values.

Our study demonstrated that Asians had a thicker postoperative SFCT than Caucasians. Just as former study showed that the black had thinner SFCT compared to the white and South Asian, the difference in ethnicities may cause this phenomenon [35].

However, in our study, DM patients were less likely to have an increase in choroid thickness. Former studies showed that diabetic patients had a thicker SFCT than normal people; even diabetic patients could have an increase in choroid thickness after intensive diabetic control, whereas no difference was observed in nonintensive diabetic control group, as choroid blood vessel circulation may be influenced by acute reduction of glycemia, which is consistent with our results [36, 37]. We could speculate that choroid thicknesses in diabetic retinopathy patients were less likely to be affected by surgery or other stimulations than choroid blood vessel circulation resulting from DM. The variation in diabetic patients was greater in absolute value than nondiabetic patients. Diabetics have a less predictable response, and therefore, the change was not statistically significant. However, choroid thickness in nondiabetic patients could be affected by cataract surgery.

The former study suggested that after surgeries, topical NSAIDs should be used in a standard way (4 to 5 times daily) for 12 weeks. If complications of cataract surgery have not been resolved, we should change the frequency of NSAIDs to a more frequent way (q1h) [38]. In our included studies, NSAIDs formulations were applied in a standard way in conjunction with corticosteroids though NSAIDs varied from flurbiprofen for 2 or 3 weeks to nepafenac for 3 weeks or 1 month. Our meta-analysis showed that differences of SFCT between preoperatively and postoperatively were not statistically significant with the use of NSAIDs, while the difference was statistically significant without the use of NSAIDs as postoperative medication. Studies also observed the complications such as keratopathy, corneal melts, and severe allergic reactions after the use of NSAIDs [39, 40]. However, these complications were really scarce, and more studies claimed that NSAIDs and corticosteroid functioned in synergy to reduce complications and increase the speed of visual recovery [41, 42].

Also, we have conducted subgroup analysis in different cataract surgery machines (Alcon and non-Alcon), different sample sizes (>50 and <50), and different OCT machines (Heidelberg and others), if OCTs were obtained with 100 times scans were used to and if OCTs were conducted in patients in the same time of a day. In Asians, for patients without NSAIDs in the postoperative medication, patients who did not use Alcon as surgery machine, patients whose averaged scans were 100 times, and patients whose OCT scans were not obtained in the same time period of a day, SFCT increased significantly at 1 month after surgery. However, SFCT was not statistically significant at 3 months. It may result from the short-term inflammatory response peaks at 1 month and data deficiencies at 3 months after surgery.

It should be noted that there were also several limitations. First, comparing with baseline values, the choroid thickness could increase in some regions or at certain timepoints after cataract surgery [3], but we only analyzed the SFCT because of the insufficiency of data measured at the temporal and nasal part. Second, the measurements of the choroid (the lines of the RPE and choroidal-scleral interface) were determined manually, which could cause errors. Third, although we had subgroup analysis in patients with variable types of OCT instruments, surgery machines, and different scan methods, we still could not control the confounding factors when we conducted some other subgroup analyses.

In conclusion, the development of OCT has helped us to understand the choroid in detail [10], and EDI-OCT has good reproducibility among choroidal thickness measurements of images [26–28, 43]. Our study demonstrated the relationship between choroid thickness and cataract surgery. Further studies are also needed to investigate if the choroidal thickness measured by EDI-OCT could reflect choroid circulation, inflammatory status, or even provide prognosis for visual acuity.

## 5. Conclusions

Our meta-analysis indicated that SFCT increased since 1 week after the cataract surgery, and the increase lasted for at least 3 months. Asians and patients without NSAIDs in postoperative medication were more likely to have a thicker SFCT after cataract surgery, whereas DM patients were less likely to increase in SFCT.

## Figures and Tables

**Table 1:** Characteristics of the included studies and quality scores.

Author	Year	Ethnicity	Design	No. of eyes	OCT	Surgery machine	NSAIDs	DM	100 times scans	Same measure time	Quality score
Asena	2017	Caucasians	Retrospective	27	Topcon	Alcon	Yes	0	No	No	8
Bayhan	2016	Caucasians	Prospective	38	RTvue-100	Alcon	No	0	No	Yes	8
Brito	2015	Caucasians	Prospective	35	Heidelberg	Alcon	Yes	35	No	No	8
Celik	2016	Caucasians	Prospective	30	Zeiss	Alcon	Yes	0	No	No	9
Falcao	2014	Caucasians	Prospective	14	Heidelberg	Alcon	Yes	0	Yes	No	8
Ibrahim	2017	Caucasians	Prospective	53	Heidelberg	NA	Yes	0	Yes	No	8
Jiang	2017	Asians	Prospective	100	Heidelberg	Abbott	No	0	Yes	Yes	8
Pierru	2014	Caucasians	Prospective	115	Heidelberg	NA	No	32	Yes	No	8
Shahzad	2017	Caucasians	Prospective	202	Topcon	Alcon	No	0	Yes	Yes	8
Wang	2016	Asians	Prospective	24	Zeiss	AMO	No	0	Yes	No	9
Yang	2017	Asians	Prospective	66	NA	NA	No	0	No	No	9
Yilmaz	2016	Caucasians	Prospective	65	Heidelberg	NA	Yes	0	No	Yes	8
Zheng	2017	Asians	Prospective	32	Heidelberg	NA	No	0	Yes	No	8

OCT indicates the machine used to do optical coherence tomography, NSAIDs indicate nonsteroid anti-inflammatory drugs, DM indicates the number of patients with diabetes mellitus, 100 times scans indicate if the OCT images were obtained from averaged 100 B-scans, same measure time indicates if the patients had OCT during the same period of time, NA indicates not available.
